# Factors Associated with Missed and Cancelled Appointments in the Endoscopy Unit: Descriptive Study

**DOI:** 10.7759/cureus.7264

**Published:** 2020-03-14

**Authors:** Reema Alnasser, Saad Alkhowaiter, Sarah Alhusaini, Badr Aljarallah

**Affiliations:** 1 Medicine, King Saud Univerisity, Riyadh, SAU; 2 Gastroenterology, King Khalid University Hospital, Riyadh, SAU; 3 Medicine, King Saud University, Riyadh, SAU; 4 Hepatology and Gastroenterology, Qassim University Medical City, Qassim, SAU

**Keywords:** endoscopy, missed, cancelled, nonattendance, appointments

## Abstract

Background and aim

Canceled and missed appointments at the endoscopy unit affect the quality of the provided services and can negatively impact patient outcomes. Assessing the association between the various factors relating to nonattendance will show whether the defective aspects are organizational or personal, which is essential to improve the quality of the healthcare system. Moreover, this study will be of value in our region due to the current scarcity of studies in the Middle East.

Methods

A descriptive study was conducted at King Khaled University Hospital in Riyadh, Saudi Arabia. A database of participants was established from those who missed/canceled their outpatient endoscopy clinic appointment; purposive sampling was applied, excluding those who are under 14 years old. Demographic data and organizational factors (e.g., referred clinic and the lead time) were collected from the patients’ files and a structured interview done by phone within 7-14 days of the missed/canceled appointment.

Results

A total of 919 endoscopy procedures were scheduled in an eight-week period, and 179 procedures were missed/canceled (19.48%); 84% were missed, and 16% were canceled. The highest percentage of the population had a high-school diploma or less. The results showed that roughly half of the patients were unemployed. More than two-thirds of the patients had undergone an endoscopy within the past year or less. The majority stated that they underwent the procedure in a different facility, which might be due to various reasons, one of which could be justified as long lead time.

Conclusion

An annual update of patients’ files is suggested. Text messages can help serve as a reminder in addition to clear appointment instructions that will aid in minimizing the absence rates. Overbooking is recommended to decrease the lead time and increase clinic efficiency. Raising patients’ awareness regarding the effect of missing appointments as well as upgrading the communication methods will assist in decreasing the rate of missed appointments.

## Introduction

Canceled and missed outpatient endoscopy appointments are an issue faced daily in the endoscopy units globally [1]. It is considered as a challenge since they affect the quality of the provided services and healthcare provider performance, and have a tremendous impact on the patients’ waiting time, as well as delaying the diagnosis of many serious conditions. Therefore, it can negatively influence the patient’s outcomes [2, 3]. A significant proportion of the missed appointments will be rescheduled eventually, thereby adding a burden to an already loaded waiting list [4]. This is an issue faced by many departments in the medical field but is more significant for colonoscopy clinics due to the high costs of procedures, which is an estimated net loss of $725 per day for the average facility [5].

At our facility, patients are referred for endoscopy by their doctor either from the clinic or in an inpatient setting. In the case of the typical outpatient scenario, the patient must present to the unit itself to confirm and book the appointment, at which time the procedure and preparations are explained. A week prior to the appointment, the patient will receive a text reminder. If the patient wants to cancel the appointment, he or she has the option of canceling either through a phone call or by presenting at the unit. 

The unit has about 20 bookings each day. Despite inconsistencies in many patients who attend their appointment at the facility, it is still generally busy, with procedures varying from gastroscopies to colonoscopies for either diagnostic and/or therapeutic reasons. 

In this study, our objectives were to detect the prevalence of missed and canceled appointments in addition to identifying the factors that contribute to this problem.

Furthermore, identifying the factors will show where the main defects are, whether they are individual, institutional, or both. Thus, a full understanding of these causes is essential and will provide us with guidance to enhance the quality of the healthcare system by minimizing the waiting and diagnosis time, and it will lead the way to a cost-effective system.

This study will not only benefit the healthcare system but it will also aid in the improvement of many healthcare systems in our region and worldwide. 

## Materials and methods

Study population and methods

This was a descriptive study conducted at the King Khaled University Hospital (KKUH) endoscopy clinic, Riyadh, from April 2017 to April 2018. The research ethics committee of KKUH approved the study. Purposive sampling was applied, and a database of participants from 5/2/2017 to 30/3/2017 was composed. All missed and canceled outpatient endoscopy appointments were included. 

A questionnaire was constructed based on factors previously studied [1-4]. A pilot study was carried out on a sample of 20 respondents; the questionnaire was modified afterward by integrating essential elements such as lead time and whether or not the patient is still symptomatic. The questionnaire is divided into five sections: (1) demographic data, which covers the basic personal data in addition to estimated drive time to the hospital; (2) past personal/family medical history, focused on the history of colon polyp or cancers in general; (3) organizational factors, including information about the referred clinic and the lead time (time between the referring clinic appointment and the endoscopy appointment), as well as the SMS reminder and the method of the explaining of the procedure; (4) personal factors, including questions about the reason for the procedure and if the patient is symptomatic. The most important question is the main reason for missing/canceling the appointment, which is categorized as a personal factor, such as a health-related issue, social matter, transportation, fear/anxiety, neglect/forgetfulness, or an organizational factor, such as a long waiting list or miscommunication. Finally, we incorporated a suggestion section to team up with the patients to find solutions to this issue.

Data collection and analysis methods

The data collection process was divided into two steps. First, the demographic data and questions in the section about organizational factors were retrieved from patients' files. Secondly, an 8-10-minute interview with the patient was conducted via phone call within 7-14 days of the missed/canceled appointment. All phone calls were carried out with hospital provided phones to keep the data private and secured. Any unanswered calls within the 14 days were excluded from the data analysis yet included in the prevalence calculations.

Data were analyzed using the Statistical Package for Social Studies (SPSS 22; IBM Corp., Armonk, USA). Categorical variables were expressed as percentages. The rate of nonattendance included all missed and canceled outpatient appointments regardless of the completeness of data.

## Results

A total of 919 endoscopy procedures were scheduled in an eight-week period; 179 procedures were missed/canceled (19.48%), of which 151 were missed, and 28 were canceled (84% and 16%, respectively). We were able to contact 109 of those patients. Table 1 illustrates the patients’ sociodemographic data. Gender was nearly equal (55% female and 45% male). The majority were married (76.1%). More than half of the patients (58.7%) were high school graduates or less. Moreover, 48.6% were housewives/husbands or retired. Out of the whole population, 25.7% reported that they live outside the city, which means they have to travel to attend appointments.

**Table 1:** Demographics of the participants ETA - estimated time of arrival

Demographic characteristics	Number	%
Gender		
Female	60	55.0
Male	49	45.0
Age		
< 20	3	2.8
20-34	18	16.5
35-44	23	21.1
45-54	26	23.8
55-64	24	22.0
> 65	15	13.8
Marital status		
Married	83	76.1
Single	21	19.3
Divorced	2	1.8
Widowed	3	2.8
Education		
High school graduate or less	64	58.7
Higher education	45	41.3
Occupation		
Employee	41	37.6
Student	15	13.8
Housewife/husband	30	27.5
Retired	23	21.1
ETA		
10-29 min	32	29.4
30-49 min	35	32.1
> 50 min	14	12.8
Out of Riyadh	28	25.7

Table 2 indicates the past medical history of the patients, as 56.9% suffered from a chronic illness such as diabetes, hypertension, hypothyroidism, or hyperlipidemia; 7.3% had mental issues. A few patients had a history of colon polyps and colon cancer (9.1% and 2.75%, respectively). Endoscopy had in the past 57.8%, and 29.2% of those had it in the past year or less.

**Table 2:** Past medical history of those who missed/canceled their appointment

Past medical history	Number	%
Patient suffering from mental issues	8	7.3
Patient suffering from chronic illnesses	62	56.9
Personal history		
Polyps history	10	9.2
Colon cancer history	3	2.7
Family history		
Polyps	1	0.9
Colon cancer	5	4.6
Family history of any type of cancer	30	27.5
Previous endoscopy		
Underwent endoscopy in the past	63	57.8
How long ago endoscopy was done		
< 3 months	8	7.3
3-6 months	8	7.3
7 months - 1 year	16	14.6
2-3 years	15	13.7
> 3 years	16	14.6

Table 3 demonstrates the organizational factors; 34.9 % of the appointments were a referral from the gastrointestinal clinic, and 23.9% from the ambulatory. The larger number of appointments were scheduled within four to six weeks of the booking (42.2%). Out of all respondents, 31.2% claimed that they did not receive an SMS reminder; 65.1% said that the procedure was explained to them, mostly a written explanation is given by the receptionist. 

**Table 3:** Organizational factors of those who missed/canceled their appointment

Organizational factors	Number	%
Referred clinic		
Ambulatory	26	23.9
Surgery	17	15.6
Gastrointestinal	38	34.9
Other	28	25.6
Type of procedure		
Gastroscopy	39	35.8
Colonoscopy	47	43.1
Both	23	21.1
Lead time		
< 1 week	5	4.6
1-3 weeks	21	19.3
4-6 weeks	46	42.2
> 6 weeks	37	34.0
Reminder and education		
Received an SMS reminder	75	68.8
The procedure was explained	71	65.1
How the procedure was explained		
Verbal	8	7.3
Written	60	55.0
Both	3	2.8
By whom		
Doctor	3	2.8
Receptionist	68	62.3

Table 4 shows the patient’s personal factors; 19.3 % of the patients underwent endoscopy due to bleeding along the gastrointestinal tract. Other reasons included abdominal pain (16.5%), inflammatory bowel diseases (14.7%), or routine screening for polyps/cancer (11.9%). Out of all patients, 62.4% had symptoms upon booking the appointment. However, 17.4% reported that their symptoms had subsided by the time of the appointment. The colonoscopy preparation was taken by 8.3% but they did not attend the appointment. Personal reasons for not attending (49.5%) mainly involved social matters, health-related issues, fear, and neglect. Organizational factors, including miscommunication and long lead time, constituted 15.6%. Appointments were missed due to multifactorial reasons by 16.5%.

**Table 4:** Personal factors of those who missed/canceled their appointment IBD - inflammatory bowel diseases; GI - gastrointestinal; GERD - gastroesophageal reflux disease

Personal factors	Number	%
Reasons for undergoing endoscopy		
Screening	13	11.9
IBD	16	14.7
GI bleed	21	19.3
GERD	12	11.0
Abdominal pain	18	16.5
Other	29	26.6
Symptom status		
Symptomatic at the time of booking the appointment	68	62.4
The symptoms subsided before the appointment	19	17.4
Completed drinking the preparation (if colonoscopy)	9	8.3
Reasons for not drinking the preparation (if colonoscopy)		
Could not tolerate it	8	7.4
Decided not to come	30	27.6
Could not retrieve it	4	3.7
Other	7	6.4
Reasons for missing/canceling the appointment		
Health-related issue	18	16.5
Social	19	17.4
Fear/anxiety	8	7.3
Neglect/forgetfulness	9	8.3
Miscommunication	12	11.0
Transportation	5	4.6
Long waiting list	5	4.6
Other	15	13.8
More than one of the above	18	16.5
Appointment status		
Missed	83	76.0
Canceled	26	24,0
Cancellation method		
Attending the clinic	24	22.0
Calling	2	1.8
Reasons for not canceling		
Busy	17	15.6
Forgot	17	15.6
Did not know how	10	9.2
Called but no one answered	18	16.6
Other	21	19.3
Rescheduled the appointment		
Yes	36	33.0
No	73	67.0

Only 23.9% of the patients did cancel, 92% of whom had to attend the clinic to cancel. The remaining 8% canceled via phone call. Thirty-three percent did reschedule their appointment.

## Discussion

The rate of nonattendance in the outpatient endoscopy clinic in this study is within the range reported in other studies (14.7% to 29%) [6-9].

A scope of multiple prior studies assessing the nonattendance rates illustrated that in California and North Carolina, the US, a total of 2,157 outpatient procedures were scheduled, from the records for these procedures, 263 nonattendances (12.2 %) were identified [3]. Another study in Valencia, Spain, found similar results, a total of 265 patients (14.7%) missed their appointment [7].

This study was mainly focused on the personal/organizational factors of nonattendance. The level of education somewhat corresponds with the nonattendance, as 58.7% were high school graduates or less compared with 41.3% who had received higher education. The results showed that roughly half of the patients were unemployed. Thus, we can partially exclude the working factor or conflict between the appointment timing and work hours [2, 5].

The rate of nonattendance of those who had polyps history was low, which might contribute to the fear of progression of polyps to malignancy. More than two-thirds of the patients had undergone an endoscopy in the past year or less, the majority of which stated that they underwent the procedure at a different facility, which might be justified as long lead time. In a previous study, long lead time was significantly associated with missed appointments [5, 7].

Even though the cancellation policy includes both attending the clinic and canceling via phone call, the latter is not actually implemented. Many patients claimed that they called but received no answer. In addition, some patients were unaware of the cancellation policy. 

This illustrates that the main defect is a systemic factor that is both organizational and individual. The low rate of cancellation can be attributed to the fact that 92.3% of those who canceled had to attend the clinic to do so, which was not feasible.

A number of solutions have been suggested to decrease the effect of the problem. Some of which are the application of pre-assessment clinics prior to the endoscopy appointment (in which the patients are provided with detailed verbal and written instructions and given the necessary preparations), enhanced communication, reduce referrals for patients with limited life expectancy and generate a hospital-specific endoscopy video tutorial [1-4]. The pre-assessment clinics have been implicated in one UK center and resulted in a significant decrease in the missed and canceled appointments from 22% to 3% [3].

Annual update of patients’ files is advised as many files had old/wrong numbers, SMS reminders, in addition to clear instructions about the appointment, and patient's awareness will help in minimizing the absence rates not only in the endoscopy clinic but rather in the facility as a whole [1, 6]. Overbooking is recommended to decrease the lead time and increase clinic performance [10,11].

There are some limitations to the study. A few of the electronic records of the patients have outdated contact information. Therefore, some of the patients who missed their appointments were not reachable. This resulted in a lack of their data in the questionnaire, although they were included in the prevalence. 

## Conclusions

In conclusion, raising patients’ awareness regarding the effect of missing appointments and upgrading the communication methods will assist in decreasing the number of missed appointments. We suggest annual update of patients’ files. Also, SMS reminders in addition to clear appointment instructions will aid in minimizing the absence rates. Overbooking is recommended to decrease the lead time and increase clinic efficiency.
